# drexml: A command line tool and Python package for drug repurposing

**DOI:** 10.1016/j.csbj.2024.02.027

**Published:** 2024-03-01

**Authors:** Marina Esteban-Medina, Víctor Manuel de la Oliva Roque, Sara Herráiz-Gil, María Peña-Chilet, Joaquín Dopazo, Carlos Loucera

**Affiliations:** a Platform for Computational Medicine, Andalusian Public Foundation Progress and Health-FPS, Seville, Spain; b Computational Systems Medicine, Institute of Biomedicine of Seville (IBIS), Hospital Virgen del Rocío, Seville, Spain; c Centro de Investigación Biomédica en Red de Enfermedades Raras (CIBERER-ISCIII), U714, Madrid, Spain; d Departamento de Bioingeniería, Universidad Carlos III de Madrid (UC3M), Madrid, Spain; e Regenerative Medicine and Tissue Engineering Group, Instituto de Investigación Sanitaria-Fundación Jiménez Díaz University Hospital (IIS-FJD), Madrid, Spain; f Epithelial Biomedicine Division, Centro de Investigaciones Energéticas, Medioambientales y Tecnológicas (CIEMAT), Madrid, Spain; g Platform of Big Data, AI and Biostatistics, Health Research Institute La Fe (IISLAFE), Valencia, Spain; h Centro de Investigación Biomédica en Red de Enfermedades Raras (CIBERER-ISCIII), U715, Seville, Spain; i FPS/ELIXIR-es, Hospital Virgen del Rocío, Seville, Spain

**Keywords:** Explainable machine learning, Drug repurposing, Omics, Mechanistic models

## Abstract

We introduce drexml, a command line tool and Python package for rational data-driven drug repurposing. The package employs machine learning and mechanistic signal transduction modeling to identify drug targets capable of regulating a particular disease. In addition, it employs explainability tools to contextualize potential drug targets within the functional landscape of the disease. The methodology is validated in Fanconi Anemia and Familial Melanoma, two distinct rare diseases where there is a pressing need for solutions. In the Fanconi Anemia case, the model successfully predicts previously validated repurposed drugs, while in the Familial Melanoma case, it identifies a promising set of drugs for further investigation.

## 1 Introduction

The development of new therapies is a slow and expensive process, with orphan drugs being a less attractive market for pharmaceutical companies. Drug repurposing, which involves finding new therapeutic uses for existing drugs, offers a potential solution to this problem [1]. By utilizing drugs that have already been proven safe in humans through previous approvals, the time and expenses involved in drug development can be significantly reduced [2].

Computational approaches play a vital role in predicting new indications of existing drugs, and, although data availability is crucial in this process, the accumulation of drug-target-disease information in databases, such as DrugBank [3], ChEMBL [4], OMIM [5], and OrphaNet [6] among others, has facilitated the development of novel methods for drug repositioning. Large-scale collections of gene expression profiles, such as the Connectivity Map (CMap) [7] or the Genotype-Tissue Expression project (GTEx) with over 17,000 samples from healthy individuals [8] have opened up opportunities for omics data exploitation in both industrial and academic settings. Transcriptomic approaches are particularly advantageous as they can be applied without prior knowledge of the disease or therapeutic mechanisms. However, when such knowledge is available, rational drug repositioning can complement data-driven approaches, providing a causal link between gene expression and functional cell behavior to predict drug-physiological interactions [9]. In this context, computational systems biology models have become essential tools for integrating our knowledge of complex cell signaling processes into further applications [10]. Mechanistic signaling models synthesize a biophysical understanding of network interactions, based on accumulated knowledge stored in pathways databases, to predict system perturbations [11].

Mechanistic modeling of signaling pathways is a powerful tool for understanding complex biological systems. By integrating data-driven approaches with quantitative relationships between phenotypes and high-dimensional feature data, mechanistic models can provide direct or indirect information about signaling and cell behavior [12]. A key advantage of mechanistic models is their ability to predict the potential consequences of gene perturbations (e.g., inhibition, knockout, drug effects) on pathway activity and downstream cellular function [9], [13], [14]. This has led to important insights into several open problems: from disease mechanisms [15], to drug mechanisms of action [11], and drug development [16], among others. However, mechanistic modeling of signaling pathways using high-throughput data is challenging due to the complexity of the data, models, and parameter estimation, as well as the need to account for feedback loops and cell heterogeneity.

One promising way to overcome the challenges of mechanistic modeling is to use machine learning (ML) techniques [17], [18]. ML can be used to predict a wide range of biological relationships, including morphology-genomic characteristics, drug-disease interactions, and drug repurposing [19], [20]. This is particularly useful in fields such as rare or emerging diseases, where there is limited knowledge. In these cases, ML can accelerate knowledge generation by reducing the dimensionality of the unknown and guiding hypothesis generation and testing following specific functional rationale [21].

In this work, we present drexml (Drug REpurposing using eXplainable Machine Learning and Mechanistic Models of signal transduction): a novel Python package that encapsulates our established rational data-driven approach to drug repurposing. The package streamlines the drug repurposing process, making it more accessible and efficient for researchers. drexml integrates the insights gained from prior studies and effectively encapsulates the methodology into a user-friendly Python package. To maintain consistent and easy-to-understand references throughout this work, we will use drexml for the software package and its capitalized form (DREXML) for the underlying methodology.

The package is based on a constructed computational collection of actionable models that describe the mechanism involved in a specific disease and a list of drug targets. The process involves collecting data on related mechanisms from existing open-access databases such as DisGeNET [22], OrphaNet [6], OMIM [5], and the Kyoto Encyclopedia of Genes and Genomes (KEGG) [12]. The information retrieved, together with the accumulated knowledge on the pathway databases, is used to produce a map of functional interaction from which mechanistic models are derived. The mechanistic map produced is then integrated into DREXML, which maps gene expression to pathway activity values, to enable the prediction of potentially causal relationships between proteins of interest, in our case targets of known drugs (KDTs) extracted from the DrugBank DB, and cell activities (circuit activity values) related to the mechanistic disease map. The methodology prioritizes KDTs with a high predictive score, dissecting the results into the specific influences that each KDT has over the different parts of the disease mechanistic map with Shapley Additive exPlanations (SHAP) [23], as well as over the disease map as a whole.

To evaluate the usability of the approach, the drexml package was put to the test in two different settings where there is an urgent need for solutions: (i) Fanconi Anemia (FA), a rare condition with limited therapeutic options, and (ii) Familial Melanoma (FM), a form of hereditary cancer. Finally, the results for both diseases are compared to two state-of-the-art drug repurposing methods: Drug Target Enrichment Set Analysis (DTSEA) [24] and Connectivity Map (CMap) [25].

## 2 Software description

**Fig. 1:**
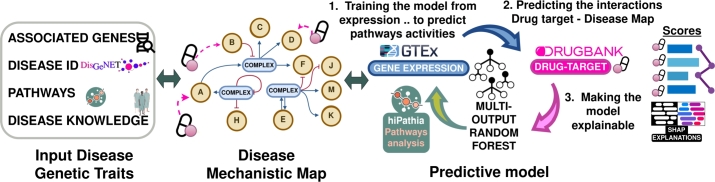
Overview of the methodology.

The drexml software is a Python package for data-driven drug repurposing. The package uses mechanistic signal transduction models to functionally describe a given disease and uses an explainable machine learning model to contextualize potential drug targets for repurposing in terms of the disease. Ultimately, the model uses the information to filter and rank the set of (gene) drug targets that can potentially regulate the disease. The package is hosted on GitHub (https://github.com/loucerac/drexml) and is available for installation using pip (https://pypi.org/project/drexml/). The GitHub repository provides documentation (https://loucerac.github.io/drexml/) and support for using the package. Fig. 1 summarizes the methodology at the core of the drexml package.

### 2.1 Software architecture

*Core concepts*  Before describing the core methodology of the drexml package, we will introduce the data concepts used:

• Known drug target (KDT): A protein or other molecule that is known to interact with a drug. In our case, we use the DrugBank database [3] (version 05.01.10) to retrieve a list of genes/proteins that are drug targets with a known pharmacological action. We refer to genes in this prefiltered list as KDTs.
• Disease map (DM): The collection of functional and directed interactions among genes, gathered in the signaling pathways, that best resembles the disease mechanism of any given disease. In our case, we decompose the signaling pathways into smaller functional units (referred to as circuits or effector subpathways in this work) using the Hipathia method [26]. The nomenclature for circuits follows the format: “Pathway Name: effector gene”. For instance, *Melanogenesis: DCT* denotes the circuit within the Melanogenesis pathway that targets the DCT gene as its effector.
• KDT expression matrix: A matrix of transcriptomic profiles of healthy samples used to infer the regulatory properties of KDTs over the signaling circuits that can be potentially altered in any given disease.
• Activity matrix: A matrix of inferred signaling activity for the same samples as the gene expression matrix. Depending on the context, we refer to the disease map as either the list of signaling circuits that compose the map or the inferred signaling activity across them.

*Precomputed data*  The drexml package automatically downloads precomputed gene and activity matrices from a Zenodo repository https://zenodo.org/doi/10.5281/zenodo.6020480. The transcriptomic samples were taken from the GTEx Portal [8] (GTEx Analysis Release V8; dbGaP Accession phs000424.v8.p2) and normalized using the edgeR package (version 3.40.0) [27]. Signaling activity was then inferred for each sample using the Hipathia package (version 2.14.0) [26], [28]. As recommended by the Hipathia authors, the activity values were normalized by the effector subpathway length. However, non-normalized pre-computed values can also be downloaded. In all cases, translation between the different gene/protein nomenclatures was carried out using MyGene [29] (version February 20, 2023).

*Using other databases and signal transduction methods*  Although the drexml package uses the GTEx and Hipathia projects as the basis for the expression and activity matrices, users can employ other databases or signal transduction methods as long as the results are in the format that drexml expects: a KDT expression matrix across a set of healthy samples and the signaling activities inferred for the same samples.

*Explainbale machine learning*

We build a multi-output random forest (MORF) [30] to predict the signaling activity throughout the disease map (the output space) using the gene expression of the KDTs as features, to infer the potential regulatory effects of the drug targets over the disease map. We use the SHAP (SHapley Additive exPlanations) values [31] to disentangle the relationships of each KDT with respect to each signaling circuit.

On the one hand, MORF regression is a type of ensemble machine learning algorithm that is specifically designed to predict multiple target variables from a set of input features. It is essentially an extension of the standard random forest algorithm for univariate responses: it works by constructing an ensemble of decision trees, where each tree is trained on a different subset of the training data. The MORF algorithm is built by aggregating multi-output decision trees, which define a split at a tree node. The criterion used to compute the split takes into account all outputs by aggregating the criterion for each output. This approach helps to regularize the split since it minimizes the criterion on all outputs.

In general, MORF regression is a powerful tool for predicting multiple target variables and can be especially useful in applications where the relationship between the input features and the target variables is complex or unclear, as in our case.

On the other hand, SHAP values are an effective instrument for explaining the predictions of machine learning models, the MORF in our case. They provide a way to understand how each feature contributes to the model output. In the case of multi-output models, SHAP values can be used to explain the predictions for each output separately. This can help to explain how the model makes different decisions based on the feature-to-output learned mappings, which in our case can be translated into disentangling the relationships between the KDTs (the features) and the signaling activity (the circuits).

More precisely, Equation (1) captures the contribution of each feature (KDT) to the prediction for each output (circuit) in a multi-output problem, providing valuable insights into the impact of individual features on the model's predictions.

(1) $$\phi_{i}^{k}=\frac{1}{K!}\sum_{S\subseteq F﹨\{i\}}\frac{|S|!(n_{circuits}-|S|-1)!}{n_{circuits}!}[f_{x}(S\cup \{i\})-f_{x}(S)]$$

where $\phi_{i}^{k}$ represents the SHAP value for feature (*i*), a KDT, and output (*k*), a circuit, $n_{circuits}$ is the number of outputs (circuits), *F* is the set of all features (the $n_{kdt}$ drug targets in our case), *S* is a subset of features, and $f_{x}(S)$ is the model's output when only the features in (S) are present.

To limit the dimensionality of the system, we select the most promising (relevant) KDTs based on the coefficient of determination ($R^{2}$ score), which measures the prediction performance. The $R^{2}$ goes from −∞, the predictions can be arbitrarily worse, to 1, a perfect prediction, where 0 is achieved when predicting the mean over the output space. The score is equivalent to the explained variance under the assumption of centered residuals.

*The method*  The DREXML methodology, described in Listing 1, takes as input the gene expression matrix *X*, the circuit activity matrix *Y*, and the user-defined thresholds: the maximum number of genes selected per circuit given by the mean absolute relevance quantile threshold ($q_{th}$), and the $R^{2}$ score threshold $R_{th}^{2}$, which can further limit the total amount of genes chosen for each circuit. The output is the signed relevance matrix $S^{\prime }$ of shape $\left(n_{kdts},n_{circuits}\right)$. The data is internally divided into *background* and validation sets, then a Multi-output Random Forest *learner* is used to fit the *background* set, and the model behavior is explained by means of computing the Shapley additive explanations (SHAP) for the *validation* set, using the *background* set to integrate them. This allows the extraction of the signed relevance of each KDT with respect to the circuits that make up the disease map. The most significant genes for each circuit (*j*) are identified using the $q_{th}$ quantile of the absolute relevances filtered by the $R^{2}$ score on the circuit *j*. Thus, the most relevant KDTs for each circuit are chosen, but the number of them is determined by the predictability of the given circuit (ill-modeled circuits result in fewer relevant genes selected). The element-wise product of the signed relevance and indicator matrices is then calculated to obtain the final result ($S^{\prime }$), also referenced as the repurposing profile through this work.

**Figure:**
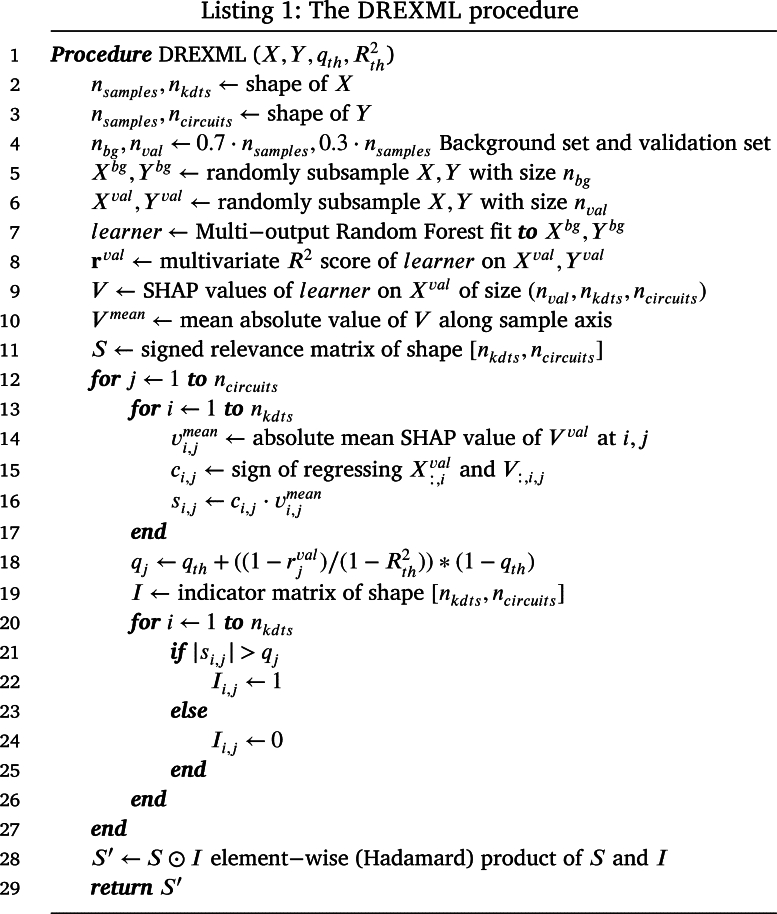

*Data-driven quality measurements*  To validate the performance of the methodology, two quality measures are used: the $R^{2}$ score and the Nogueira stability test [32]. The $R^{2}$ score measures how well the model maps the gene and signaling spaces, while the Nogueira stability test ensures that the feature selection procedure is consistent.

To control for variability in the measures, we follow the resampling strategy proposed in [32]: the samples are split into training and test sets, the procedure is fit to the training set, and its performance is assessed using the $R^{2}$ score of the test set. This process is repeated for 100 bootstrap samples of the dataset.

The result is a distribution of $R^{2}$ scores and the stability estimate. Point estimates and 95% confidence intervals for the $R^{2}$ and stability measures are then reported for each circuit and as a map-wise average.

*Hyperparameters*  The learner hyperparameters are set to 200 multi-output regression trees with a *maximum depth* of 8, which are trained on partitions of up to $\mathrm{ceil}(\sqrt{n_{kdts}}+20)$ genes. The mean squared error (MSE) is used to evaluate the quality of each split. To speed up the process, SHAP values are integrated with a subsample (n=1000) of the background set, following the guidelines of the Python shap package (v 0.42). The hyperparameters for feature selection are set to a $R^{2}$ threshold ($R_{th}^{2}$) of 0.5 and a quantile threshold ($q_{th}$) of 0.95.

*Hyperparameter tunning*  The drexml package allows users to fine-tune the tool for specific datasets by adjusting the –n-iters parameter. The process involves trying different configurations (parameter combinations), starting with minimal resources and gradually increasing them for promising candidates. These *candidates*, the solution to the optimization problem, are different versions of the MORF *learner* with varying configurations, namely the maximum depth and number of features used by each multi-output regression tree. By adjusting –n-iters to an integer greater than 0, the user sets the maximum amount of resources (trees) allocated to each candidate, ensuring efficient use of computational power. The procedure follows the Successive Halving search strategy [33] as implemented in the scikit-learn library [34].

*Imputation*  In the case that the data provided by the user contains missing values, the package automatically encloses the learner in a pipeline that first imputes the data (note that the drexml package assumes normalized gene expression matrices). It works by identifying the *k* nearest neighbors for each data point with missing values, using the available features. This ensures that the imputed values are consistent with the overall data distribution. The procedure follows the k-Nearest Neighbors imputation strategy [35] as implemented in the scikit-learn library [34].

### 2.2 Software implementation

The Python package is compatible with Python versions from 3.8 to 3.10. The package is implemented as a command-line interface (CLI) program that can be installed using the Python Package Index (PyPI) with pip install drexml.

Machine learning and scientific functionalities have been implemented using the Scikit-learn [34], NumPy [36], and SciPy [37] Python libraries. Model explainability is implemented using TreeShap [31], a version of SHAP specialized for tree-based learners. The installation process can be tuned to trigger the installation of the GpuTreeShap GPU extension to take advantage of the CUDA-accelerated version of the algorithm [38]. To install the tools needed to compile the extensions, we recommend using the conda (https://docs.conda.io/) package manager using the instructions found in Listing 2.

**Figure:**
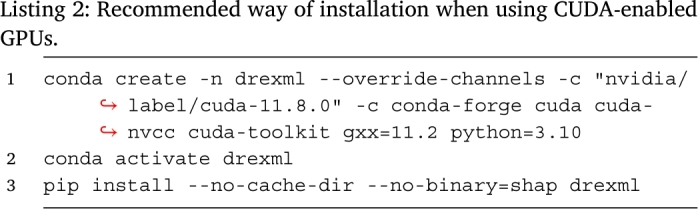

To use the parallel processing capabilities of the drexml package, set the –n-gpus or –n-cpus parameters, or both, to an integer greater than 1. If either parameter is set to -1 or lower, the program will use all available CPUs and/or GPUs. The package uses the parallel capabilities in two scenarios. On the one hand, all learners are fitted on the CPUs using as many processes as specified by the –n-cpus parameter. On the other hand, the software splits the *validation* set (see Listing 1) into as many batches as either the number indicated by the –n-cpus (if GpuTreeShap is not available or –n-gpus is set to 0) or –n-gpus parameter (if –n-gpus is set to an integer different than 0 and the GpuTreeShap extension is available). Then, each batch is computed in parallel using one device per process. To turn off CUDA-based computations even when GpuTreeShap is available, set –n-gpus to 0.

The main entry point to use the package is the run subcommand and the disease definition file path. With “drexml run disease.env” the software executes the full validation procedure and a final fit of the methodology according to the variables defined in disease.env (see the next section).

Other less important CLI arguments can be used as described in the documentation, which can be accessed in https://loucerac.github.io/drexml/cli-reference.html. It should be noted that the drexml package is also a library written in pure Python, so all of its components can be imported in the usual manner.

*The disease definition file*  The software is primarily interacted with via the disease definition file, which is a simple text file that follows the well-known environment specification file format. The variables included in the file can be divided into three categories: those that relate to the construction of the disease map and activity matrix, those that relate to the KDT gene space, and those that specify versions (see subsection 2.4 for examples).

The variables related to the disease map and the activity matrix include seed_genes, disease_id, use_physio, pathvals, circuits, activity_normalizer, and circuits_column. The seed_genes, disease_id, and circuits variables are complementary, collectively contributing to automatically building the disease map. The seed_genes variable expects a list of Entrez identifiers that are suspected to be related to the disease. The algorithm searches the genes across signaling effector sub-pathways (using the KEGG database) and returns the list of those that contain at least one seed gene. The disease_id variable expects a disease identifier (using UMLS CUI identifiers), and then the package looks at the genes associated with the disease using the curated DISGENET database (automatically retrieved by the package) and uses them as the seed genes. Finally, the circuits variable expects a list of circuit names or a path to a TSV file where the circuits to use are stored in a column named after the contents of circuits_column using binary values, and each row is indexed using the circuit names. The use_physio variable is a binary variable that informs the algorithm to use only the physiological pathways: a curated list of signaling pathways that do not contain known disease-specific pathways. If set to true, the list of circuits is trimmed down by keeping only those that are contained in the physiological list. The list can be retrieved from the circuit_names.tsv.gz file in the package resources folder. The activity_normalizer variable expects a binary value; if set to true then the package uses the length-normalized activity matrix.

The variables that deal with the KDT gene space include gene_exp, genes, and genes_column. The genes variable stores the gene metadata in the form of a three-column TSV file with the Entrez identifiers (entrez_id column), symbol_id (the HUGO symbol translation), and a binary column indicating if the gene is KDT or not (named after the contents of the genes_column variable).

If the user wants to use a different signal transduction algorithm and/or a different database of transcriptomic profiles to infer the signaling activity, they can utilize the pathvals and gene_exp variables. The pathvals variable provides a path for the signaling activity matrix, while the gene_exp variable provides a path for the gene expression matrix.

The variables that specify versions include GTEX_VERSION, MYGENE_VERSION, DRUGBANK_VERSION, HIPATHIA_VERSION, and EDGER_VERSION. These variables are used to tell the software to download a specific set of data. The available versions can be checked in the Zenodo data repository for the package https://zenodo.org/doi/10.5281/zenodo.6020480. Note that even if new versions of GTEx (or any of the other data sources) are made available by their respective owners but the Zenodo repository is not consistently kept up-to-date, a user can always build all data resources and run the algorithm without resorting to the provided data.

### 2.3 Package functionalities

The main function of the software is to run the DREXML procedure for a given disease map. Most users will use the default databases and one of the map construction methods described above. To trigger the procedure, users can run the following command “drexml run disease.env” where disease.env is the specification file described in the previous section.

The results of the operation are stored as TSV files: i) the scoring matrix containing the $R^{2}$ and stability estimates along with their confidence intervals for the disease map and each of its circuits, and ii) the final relevance matrix $S^{\prime }$ (i.e. the repurposing profile for the disease under study).

**Fig. 2:**
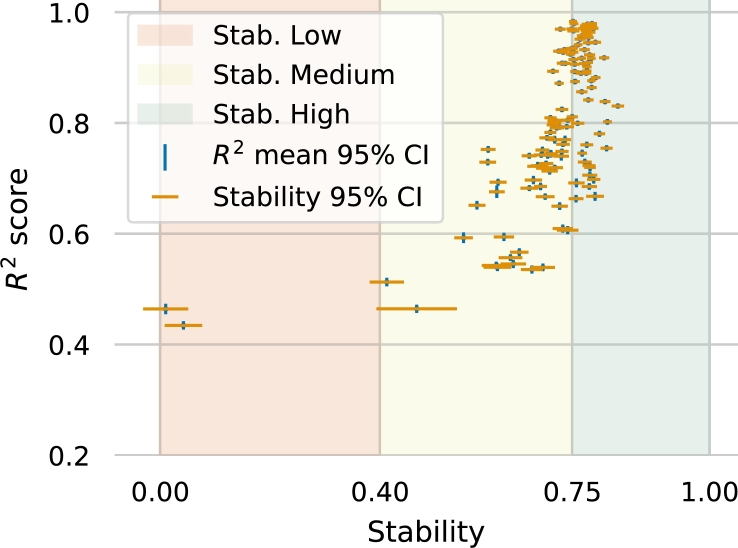
Stability and *R*^2^ performance for the Fanconi Anemia example. This graphic uses color to represent stability, with red indicating poor stability, yellow indicating medium stability, and green indicating high stability.

The plot subcommand allows users to generate summary graphics of the stability and results. For example, Fig. 2 shows the summary results for the Fanconi Anemia example (see subsection 3.1 for more details). The x-axis of the plot shows the stability statistic, while the y-axis displays the $R^{2}$ score distribution across the test folds. Color bands represent the effect size of the stability score according to [32]. Let *s* be the stability estimate: the color red indicates a poor stability performance (0<s≤0.4), yellow indicates a mid-quality performance (0.4<s<0.75), and green indicates an excellent stability performance of the feature selection procedure (0.75≤s≤1).

Additionally, the plot subcommand generates a cluster map of the relevances similar to those shown in section 3. The x-axis of the cluster map represents the KDT genes, while the y-axis depicts each of the circuits that make up the disease map under study. Each cell of the cluster map displays the relevance of each KDT-circuit pair. Thus, each column of the map shows the contextualization of the KDT in terms of the mechanistic map.

Moreover, users can use the option –gene GENE to plot a specified gene (GENE) in more detail, i.e. it displays the corresponding column of the relevance map.

Note that by default, nonselected KDTs are omitted. Additionally, circuits where no KDT has been selected or where the lower confidence interval for the stability estimate is poor (below 0.4) are also excluded.

All graphics are saved in high-quality, publication-ready file formats (PNG and PDF).

### 2.4 Sample code snippets

This section describes four use cases for drexml.

*Use case 1*  In Listing 3 the user declares in file disease_01.env that a disease map should be constructed from mapping the genes provided in seed_genes (*Entrez* format) to the KEGG effector circuits, discarding all disease-specific pathways, i.e. using only those pathways categorized as *physiological*, (setting use_physio to true), with its activity normalized by the circuits-length (setting activity_normalizer to true). Note that the list of physiological pathways can be found in the resources/circuit_names.tsv.gz file.

**Figure:**
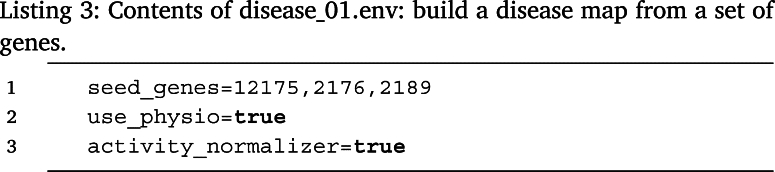

*Use case 2*  In Listing 4 the user declares in file disease_ulms.env that a disease map should be constructed from mapping the genes associated with the disease defined by the identifier C0015625, which corresponds to Fanconi Anemia in the Unified Medical Language System (ULMS), to the KEGG effector circuits using all the available pathways (setting use_physio to false).

**Figure:**
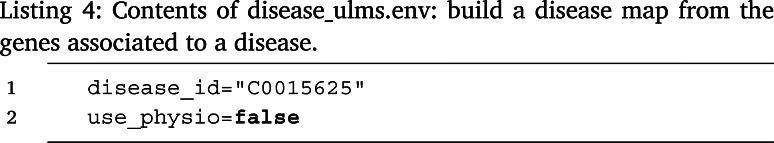

*Use case 3*  To use a disease map that is completely contained in KEGG the user needs to provide either the circuit names or their identifiers (as defined in the Hipathia package [26]). See Listing 5 for an example environment file (disease_02.env) where the user specifies a map composed of two circuits (using the Hipathia identifiers).

**Figure:**
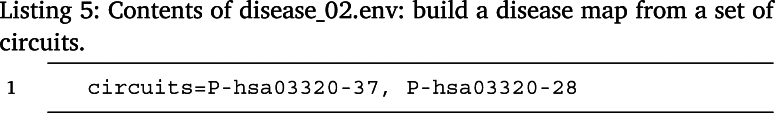

Note that file circuits2genes_gtex-v8_hipathia-v2-14-0.tsv.gz in https://zenodo.org/doi/10.5281/zenodo.6020480 contains a complete mapping between the identifiers and the human-readable names.

*Use case 4*  In the previous use cases, the model employs a precomputed circuit activity matrix that encompasses the KEGG signalization pathway database (using Hipathia R package v2.14.0). However, the user can provide its own activity matrix, usually due to having a more concise map about the disease under study to what is already available in KEGG. To do so, the only requirement is to set the environment variable pathvals to the path where the results should be stored in TSV, TSV.GZ, or Feather format with the circuit names or identifiers in the columns and the sample names in the index. If a gene expression matrix is not provided (setting the gene_exp environment variable to the path where the dataset is stored), the package assumes that the user wants to employ the precomputed GTEx dataset. Note that the gene expression follows the same format as the activity matrix: a TSV, TSV.GZ or Feather file with genes (Entrez identifiers) as columns and samples as rows. Listing 6 contains a disease environment file where the user provides both matrices.

**Figure:**
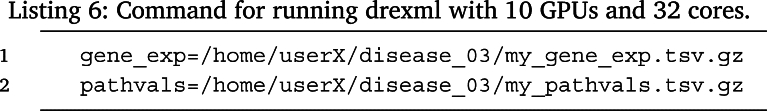

Finally, in order to run either of these examples, the user only needs to execute the run sub-command from the drexml package pointing to the disease environment path. For example, to run the experiment associated to the environment file named disease_01.env as described in Listing 3 using 10 GPUs and 32 cores, the user needs to execute Listing 7.

**Figure:**
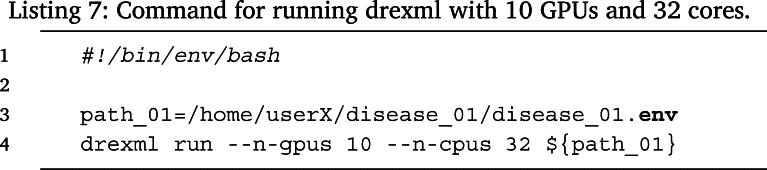

Instead, Listing 8 shows how to run the experiment with all the available resources.

**Figure:**
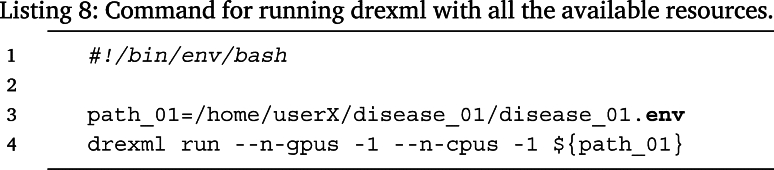

### 2.5 Package runtimes

**Fig. 3:**
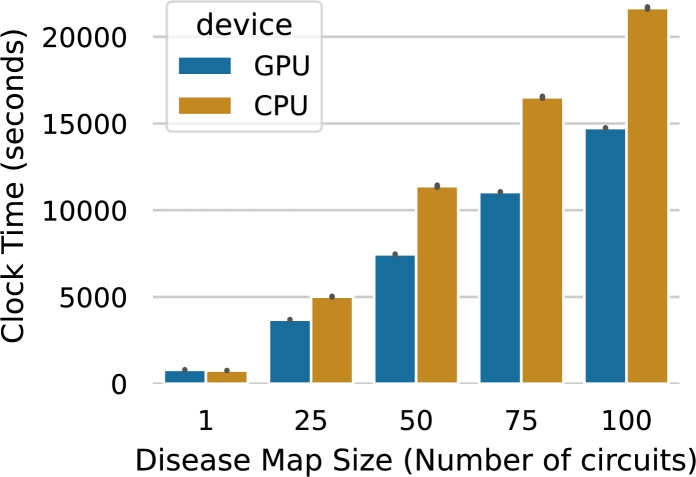
Benchmarking the clock time (in seconds) spent when running drexml for different disease map sizes on different devices (CPU/GPU).

In this section, we conduct tests to measure the memory consumption and runtime performance of the drexml package on disease maps of varying sizes. We simulate disease maps by randomly selecting 1, 25, 50, and 100 circuits, representing small to large-scale scenarios. To account for potential variability, each disease map size was run ten times using Hyperfine software (v1.18.0) [39] on both CPU (32 Intel(R) Xeon(R) Gold 6230R @ 2.10GHz) and GPU (32 Intel(R) Xeon(R) Gold 6230R @ 2.10GHz with 3 Nvidia (R) V100 32 GB) hardware. Fig. 3 shows benchmarking results that highlight the significant advantages of using GPU hardware compared to traditional CPUs for processing large-scale disease maps with drexml.

Table 1 for a tabular representation of the *mean* and *standard deviation* of the runtime measured in seconds for GPU and CPU systems. The table also shows the maximum RAM and GPU-RAM used during the benchmarking.

**Table 1:** Memory (in GB) and clock time benchmarking results (in seconds) for the drexml package on varying disease map sizes (number of circuits) and hardware configurations (CPU/GPU).

	mean		stddev		max_memory_cpu		max_memory_gpu	
Device	CPU	GPU	CPU	GPU	CPU	GPU	CPU	GPU
Map size								
1	756.09	799.64	2.10	2.14	9.86 G	6.76 G	-	0.41 G
25	5015.78	3692.54	28.54	8.38	17.10 G	10.9 G	-	1.82 G
50	11384.62	7456.56	63.46	14.10	28.22 G	14.18 G	-	3.91 G
75	16514.22	11050.92	60.96	17.46	34.57 G	16.5 G	-	4.2 G
100	21658.04	14736.68	70.05	19.63	38.16 G	18.5 G	-	5.4 G

The code used to conduct the benchmark can be found in the following folder of the GitHub repository: drexml/examples/benchmark.

### 2.6 Software reproducibility

In addition to the code examples referenced in Section 3, we have created a minimal working example available as a Code Ocean reproducibility capsule accessible at https://doi.org/10.24433/CO.8171877.v1 (estimated runtime: 24 minutes.)

## 3 Results and discussion

In this section, we present the results of our drug repurposing studies for Fanconi Anemia (FA) and Familial Melanoma (FM) using the drexml package. The FA use case demonstrates how the improved algorithms produce more fine-grained results than those in the previous study [40], which used a predecessor of the methodology. Note that this is the first time the software has been made available. In the FM case, this is the first time the methodology has been applied.

For the Fanconi Anemia (FA) analysis, the DisGeNet database was queried using the disease identifier “C0015625” to extract gene seeds for the drexml tool. Conversely, the Familial Melanoma (FM) analysis utilized ORPHANET-associated genes as the model's seed input.

### 3.1 Fanconi anemia

**Fig. 4:**
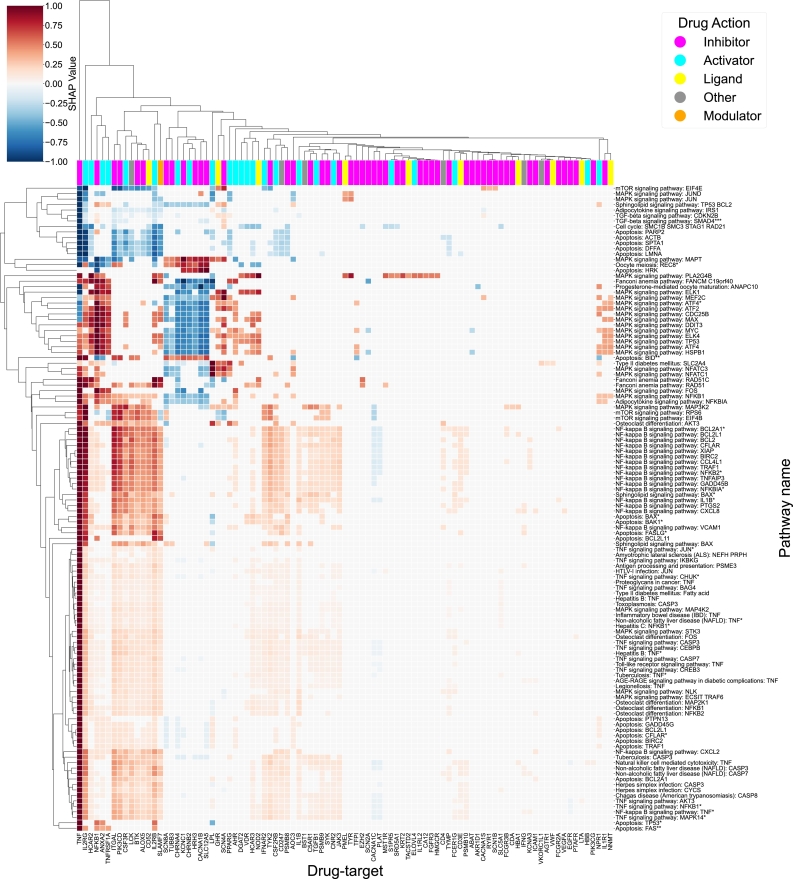
Fanconi Anemia repurposing profile: Heatmap of the repurposing profile *S*′ for Fanconi Anemia. *S*′ is scaled to (−1,1) on a per-circuit basis: KDTs are on the x-axis and circuits on the y-axis. The sign indicates the direction of the KDT influence over each specific circuit and the score value depicts how strong is the influence of a specific KDT for predicting the activity of a specific circuit. The top color bar represents the most frequent drug effects of the drugs targeting that specific KDT.

Fanconi Anemia (FA) is a rare genetic disorder that affects the body's ability to repair DNA damage. FA can lead to bone marrow failure, leukemia, and solid tumors. The initial application of the drexml package to Fanconi Anemia (FA) has revealed potential alternative drug targets that could guide drug repurposing efforts. We used the drexml package with the environment defined in Listing 4 and the other parameters set to their default values. Fig. 4 shows a heatmap representation of the relevance scores ($S^{\prime }$) that delineates the repurposing profile for the FA disease map, with known drug targets (KDT) in the columns and FA circuits in the rows. In particular, high-scoring KDTs such as TNF, NFKB1, and PIK3CD have a significant alignment with the pathophysiology of FA, making them attractive therapeutic targets.

The Fanconi Anemia pathway is a key component in understanding the mechanisms of the disease. The pathway is essential in the maintenance of the genome, coordinating intricate DNA repair mechanisms [41]. Dysfunction in this pathway in FA patients results in various malignancies, including leukemia and solid tumors [42], [43].

DNA damage, when left unrepaired, can culminate in cellular senescence or even cell death. The cytokine TNF, pivotal in systemic inflammation, can act as an inducer for cell death. Within the FA context, a compromised FA pathway can result in persistent DNA damage, potentially amplifying TNF signaling [44].

Parallelly, the NF-*κ*B pathway, with NFKB1 at its core, is known to be activated in response to various stress stimuli, DNA damage being a primary one. Given the backdrop of FA, where DNA remains unrepaired, sustained activation of the NF-*κ*B pathway is plausible [45]. The PI3K/AKT signaling pathway, where PIK3CD stands as a central figure, is essential for cellular processes such as survival, growth, and proliferation. DNA damage can trigger this pathway, serving as a survival mechanism. However, the inherent DNA repair defects associated with FA can lead to chronic activation of the PI3K/AKT pathway, facilitating unchecked cell proliferation, a defining trait of cancer [46].

Nevertheless, targeting proteins that play central roles in a multitude of cellular processes presents a double-edged sword. While these proteins, such as TNF, NFKB1, and PIK3CD, may appear attractive targets due to their broad influence on the disease pathophysiology, their ubiquitous involvement raises concerns about potential off-target effects and unintended consequences. This risk is heightened in FA patients, who are inherently prone to DNA damage and often cannot tolerate conventional treatments like chemotherapy and radiation [47]. Thus, guided non-genotoxic therapies are necessary for these patients.

In this sense, TGF*β*1 and EGFR emerge as more promising candidates, offering a narrower and potentially safer therapeutic window. TGF*β*1, part of the transforming growth factor-beta family, has been intricately linked with the bone marrow failure observed in FA. The hyperactive growth-suppressive TGF*β*1 pathway in FA, regulated by TGF*β*1 and its associated ligands, presents a clear therapeutic target.

Specific inhibitors of TGF*β*1, such as AVID200, have shown potential in promoting the survival of FA hematopoietic stem and progenitor cells (HSPCs). Notably, these inhibitors have demonstrated the ability to downregulate DNA damage pathways, further underscoring their therapeutic potential [48].

The epidermal growth factor receptor (EGFR) plays a pivotal role in cell growth and differentiation. Its specific involvement in certain cancers, particularly head and neck squamous cell carcinoma (HNSCC) associated with FA, makes it an attractive target. Drugs inhibiting EGFR, such as gefitinib and afatinib, have shown remarkable specificity in their action, displaying significant cytotoxicity against FA HNSCC cell lines without adversely affecting the FA signaling pathway or inducing chromosomal fragility [49]).

In this case, as shown in Fig. 4, only four circuits are affected by drugs targeting EGFR (the y-axis), which are all associated with TNF signaling. These circuits are all negatively correlated with the EGFR KDT (i.e. low expression of EGFR potentially raises the circuit activity).

While the role of TNF in carcinogenesis is complex [44], [50] and this transcription factor influences a wide array of downstream processes [51], the success of EGFR targeting drugs in the treatment of FA associated HNSCC could partially be due to its influence over certain TNF-related circuits.

More importantly, according to the model, EGFR as a drug target does not affect any of the Fanconi Anemia pathway circuits, explaining its nontoxic effect on non-cancerous Fanconi Anemia deficient cell lines and mice, while still promoting cytotoxicity, apoptosis, and tumor suppression in the context of the disease. This behavior corresponds directly to the constraints imposed in [49] when searching for repurposable drugs in FA.

Furthermore, we observe that among the drugs explored, inhibition is the main drug effect associated with EGFR. Here, the drexml package allows us to gain evidence and narrow the window of the hypothesis to test and verify with experimental observations.

In this same light, other drug targets shown to influence carcinogenesis-related circuits, while not affecting the Fanconi Anemia pathway itself, could be explored to treat other Fanconi Anemia-related malignancies. Such is the case with LTA [52] or GHR [53]. In this case, the package allows us to generate a hypothesis on the influence these drug targets may have over FA, which could be used to inform future experimental setups.

**Fig. 5:**
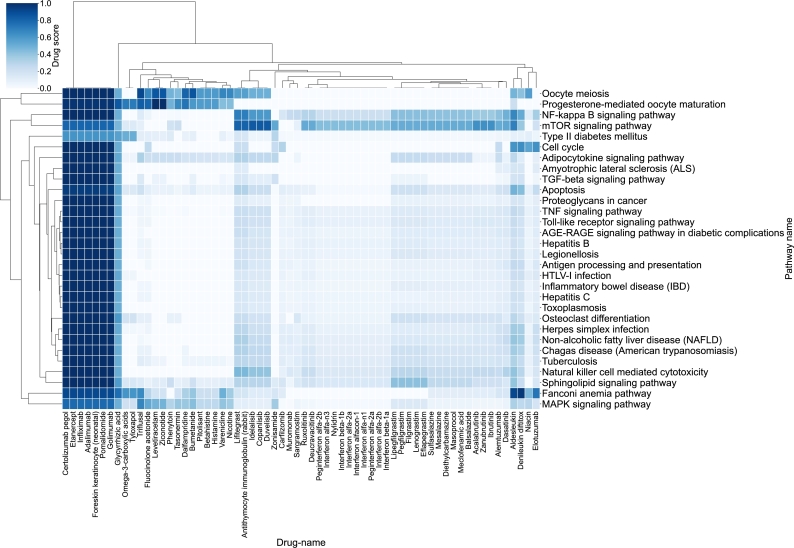
Heatmap of the 30 highest-ranked drugs according to their *ℓ*_1_ norm, grouped by pathway. Each cell represents the aggregated relevance of a given drug across the stable circuits that compose each pathway represented in the FA disease map.

The same rationale can be explored at the drug level. In this sense, Fig. 5 shows the main drugs that affect the pathways involved in Fanconi Anemia. Here, we observe that various drugs show a strong effect on the NF-kappa B signaling pathway, while showing a weak association with other pathways, importantly, the Fanconi Anemia pathway. Complementing EGFR inhibition, these drugs could be further explored for the targeted inhibition of this pathway in the case of Fanconi Anemia.

Although an in-depth analysis of the potential of these drug targets for the treatment of Fanconi anemia-related malignancies is beyond the scope of this example of use, the drexml package could also allow the study of the effect of these targets on circuits related to genome stability as a whole. This would add to the case that these targets need to be further studied as a treatment for Fanconi Anemia-related malignancies.

A reproducible example can be found in the following folder of the GitHub repository: drexml/examples/fanconi_anemia.

### 3.2 Familial melanoma

**Fig. 6:**
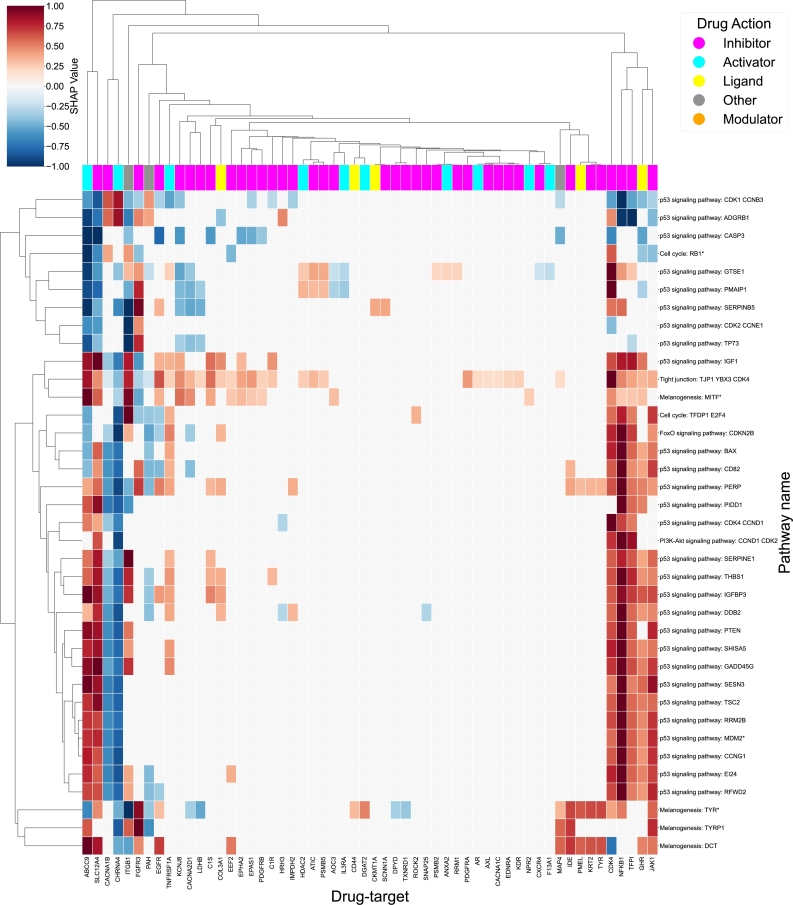
Familia Melanoma repurposing profile: Heatmap plot of the normalized (-1,1) SHAP scores from the predictive KDTs (X-axis) over the stable circuits (Y-axis) of the FM disease Map. The sign indicates the direction of the KDT influence over each specific circuit and the score value depicts how strong is the influence of a specific KDT for predicting the activity of a specific circuit. The top color bar represents the most frequent drug effects of the drugs targeting that specific KDT.

As a second example, we have applied the drexml package on Familial Melanoma (FM), a rare disease caused by inherited genetic mutations that ultimately trigger melanoma, a type of skin cancer [54]. As depicted in the rows of Fig. 6, the FM disease map consists of the circuits that contain a gene associated with FM. Here, most circuits correspond to the p53 signaling pathway, which is primarily responsible for tumor suppression and genome integrity, and is known to be involved in a large number of cancers, including skin cancer [55].

Here, various KDTs such as CDK4 or JAK1 show a high influence on the FM disease mechanism, as they are predicted to affect most FM circuits. Dysregulation of the p16ink4A-cyclin D-CDK4/6-RB pathway is prevalent in many cancer types and is observed in up to 90% of melanomas. Specifically, mutations in CDK4, particularly at the arginine residue 24 (CDK4R24C), have been identified in melanoma patients with a familial predisposition [56].

Furthermore, the JAK-STAT pathway plays a crucial role in various cellular processes, including cell growth, differentiation, and immune responses. In the context of melanoma, the JAK-STAT pathway, and JAK1/2 in particular, have received significant attention due to their implications in disease progression, immune evasion, and therapeutic resistance. In melanomas resistant to immune checkpoint blockade (ICB), a surprising discovery was the aberrant sustained activation of the mTOR-JAK1/2 axis, centered around JAK1/2 in tumors lacking IFN-*λ* signaling [57].

However, more specifically for melanoma is the involvement of various circuits belonging to the melanogenesis pathway. Exploiting the relationship of KDTs with these circuits could lead to lineage-specific adjuvant therapies for melanoma.

Although melanin is crucial in photoprotection against genotoxic effects of ultraviolet radiation [58], high concentrations of intracellular melanin within melanocytes have been associated with resistance to current therapies for primary and metastatic melanoma [59], [60], and deterioration of overall patient survival [61]. In fact, depigmentation of melanoma cells has been found to increase their photodynamic and chemotherapeutic sensitivity [62], [63], [64]. Depigmented melanoma cells showed a vastly greater sensitivity to lymphocyte-mediated cytotoxicity [64].

As such, while the depigmentation of melanoma cells through the inhibition of melanin biosynthesis, i.e. melanogenesis has been poorly explored in clinical applications, initial results show its potential as a targeted therapy for melanoma. [65].

Through the repurposing profile, we observe several circuits with their drug targets specifically involved with these melanogenesis signaling circuits, namely: CD44, DGAT2, DPYD, and NPR2. Accordingly, we observe that KDTs such as CD44 and DGAT2 positively correlate with the Melanogenesis: TYR circuit. Indeed, the inhibition of human Tyrosinase (TYR) has been put forward as a potential target in melanoma management [65].

In this sense, the inhibition of these positively associated KDTs could lead to an indirect inhibition of TYR at the signaling regulatory level, while potentially not compromising a large number of other circuits leading to unwanted side effects.

Furthermore, the predicted targeted inhibition of CD44, depicted in Fig. 6, for specific circuits, in and of itself has been put forward as a potential target in various cancers [66]. In this same light, the selective promotion of the negatively associated DPYD could have this same desired inhibitory effect. Furthermore, an elevated expression of DPYD is positively correlated with radio and chemotherapy response in rectal cancer [67], leading to the belief that its promotion does not have an oncogenic effect.

**Fig. 7:**
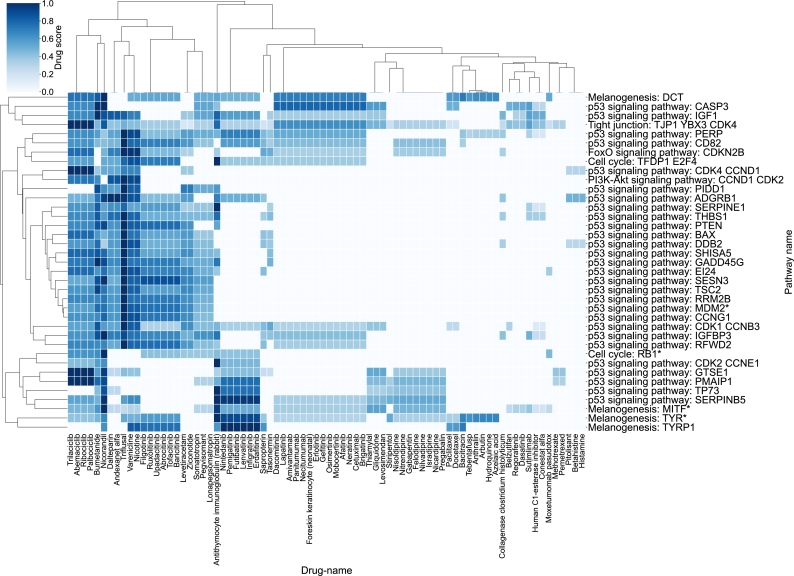
Familial melanoma repurposing profile of drugs to pathway: drugs targeting the KDTs (X-axis) with the top 30 highest absolute SHAP scores on any given stable circuit of the FM disease map (Y-axis). Drugs were obtained from DrugBank v5.1.10.

Finally, these associations can be investigated at the drug-to-pathway level, as illustrated in Fig. 7. This heatmap shows the drugs targeting the top 30 highest-scored KDTs associated with any given FM circuit on the columns and, on the rows, the pathways to which the circuits belong.

For instance, drugs such as Paclitaxel, Docetaxel, and Moxetumomab pasudotox target specific melanogenesis circuits. Notably, all of these drugs are currently approved chemotherapeutic agents used to treat various cancers. Therefore, their potential inclusion in melanoma treatment could be explored based on the insights provided by the drexml tool.

Finally, ruxolitinib, a JAK1/2 inhibitor, emerges as another promising drug candidate. Its repurposable profile can be explored in Fig. 7, and experimental findings from the literature further support the predictions obtained. For example, a study demonstrated that Ruxolitinib treatment effectively and selectively suppresses ICB-resistant melanoma cells [57]. This approach holds promise as a personalized therapeutic strategy for ICB-resistant melanomas.

Here again, drexml package allows to generate hypotheses that can pave the way for more precise targeted experiments, boosting the discovery of new potential therapeutic targets.

A reproducible example can be found in the following folder of the GitHub repository: drexml/examples/familial_melanoma.

## 4 Comparison with other methodologies

To assess the performance of the drexml method in drug repositioning, we compared the overlap of its predictions with those of two other state-of-the-art network-based methods: cMAP [25] and DTSEA [24]. We focused on two specific diseases, Familial Melanoma (FM), and Fanconi Anemia (FA), to enable a direct comparison with our drexml analysis.

To facilitate a direct and fair comparison with drexml, we employed the same seed genes for each analysis. For the FA use case, we selected 25 genes from the DisGeNet database with a gene-disease association score (GDS) of 0.3 or higher. In the FM use case, we utilized genes associated with FM in the ORPHANET database, resulting in a set of 11 seed genes. The code can be found in the following folders of the GitHub repository: drexml/examples/DTSEA_comparison and drexml/examples/cmap_pertubagens_comparison.

### 4.1 DTSEA analysis

The Drug Target Set Enrichment Analysis (DTSEA) method stands out for its network-based approach to drug repositioning. It maps disease-related genes into a gene functional interaction network and uses the Random Walk with Restart (RWR) algorithm to compute the proximity between disease genes and drug targets. By applying the GSEA (Gene Set Enrichment Analysis) approach, DTSEA calculates the enrichment score (ES) for each drug, reflecting its mean distance to the disease in the network. This method prioritizes drugs according to their normalized enrichment scores (NES) and p-values, identifying those with a closer network proximity to disease-related genes [24]. The DTSEA method also relies on the DrugBank database, making comparisons easier.

#### 4.1.1 DTSEA: fanconi anemia

Results from the Fanconi Anemia comparison show a degree of overlap of 11.5% of DTSEA-relevant drugs on the drexml predictive drug targets and a complementary 6.40%. The overlapping targets, sorted by their p-value and NES (Normalized Enrichment Score), as depicted in Table 2, show a significant variation in terms of their importance. The comparative analysis highlights EGFR, TUBB3, and VEGFA with relatively high significance based on their p-values and NES scores, indicating strong drug-target predictions within the DTSEA dataset, while exhibiting specificity for a mild number of circuits, with modest DREXML scores. In contrast, TNF, as well as PIK3CD, show the highest variance in DREXML scores, with both negative and positive extremes, indicating their variable and highly regulatory role across many pathways, which rather than promising, could present undesired effects due to their ubiquity.

**Table 2:** Drexml - DTSEA overlapping drug-target interactions for FA (Fanconi Anemia) and FM (Familial Melanoma).

Target	Drug ID	Drug Name	FDR p-value	NES	R.NES	DREXML	Circuits	Dis.
PIK3CD	DB11891	Fimepinostat	5.864× 10^−5^	2.352	17	0.256	88	FA
EGFR	DB12174	CUDC-101	1.070× 10^−4^	2.310	18	0.002	4	FA
VEGFA	DB10772	Foreskin keratinocyte (neonatal)	6.059× 10^−4^	2.250	22	0.001	1	FA
TNF	DB05992	Plinabulin	1.806× 10^−3^	2.179	34	0.929	117	FA
EGFR	DB03496	Alvocidib	1.922× 10^−3^	2.139	43	0.002	4	FA
LPL	DB13751	Glycyrrhizic acid	2.994× 10^−3^	1.989	80	0.119	41	FA
TUBB3	DB04845	Ixabepilone	3.973× 10^−3^	2.127	46	0.059	19	FA
VEGFA	DB05294	Vandetanib	6.768× 10^−3^	2.060	65	0.001	1	FA
LDHB	DB11638	Artenimol	1.984× 10^−7^	2.402	28	0.053	5	FM
RRM1	DB00631	Clofarabine	2.693× 10^−7^	2.497	20	0.006	1	FM
EGFR	DB03496	Alvocidib	7.079× 10^−7^	2.411	26	0.170	14	FM
PSMB2	DB11762	Marizomib	1.608× 10^−6^	2.500	19	0.006	1	FM
PDGFRB	DB09283	Trapidil	3.652× 10^−6^	2.420	24	0.024	3	FM
AR	DB00421	Spironolactone	1.032× 10^−4^	2.250	66	0.005	1	FM
EGFR	DB12174	CUDC-101	1.232× 10^−4^	2.207	72	0.170	14	FM
KDR	DB05608	MKC-1	1.023× 10^−3^	2.157	80	0.007	1	FM
PDGFRB	DB12147	Erdafitinib	1.183× 10^−3^	2.098	94	0.024	3	FM
CACNA2D1	DB00230	Pregabalin	1.230× 10^−3^	2.091	97	0.101	9	FM
CACNA2D1	DB08872	Gabapentin enacarbil	1.230× 10^−3^	2.091	98	0.101	9	FM
AR	DB08804	Nandrolone decanoate	1.956× 10^−3^	2.088	104	0.005	1	FM
PDGFRB	DB11694	Ilorasertib	3.465× 10^−3^	2.003	135	0.024	3	FM
AR	DB00717	Norethisterone	7.143× 10^−3^	1.664	238	0.005	1	FM
AR	DB08867	Ulipristal	7.143× 10^−3^	1.664	239	0.005	1	FM
AR	DB14583	Segesterone acetate	7.143× 10^−3^	1.664	240	0.005	1	FM

This comparative analysis elucidates the nuanced roles of overlapping targets in both datasets. Interestingly, the DTSEA results highlight EGFR, and TUBB3 for their network proximity to disease genes and significant NES and p-value rankings, while drexml further contextualizes these targets within biological pathways, offering insights into their mechanistic roles in disease modulation. As depicted in Table 2, the number of circuits that these targets influence showcases their specificity. This congruence underscores their potential utility in therapeutic interventions.

The variability and context-dependent significance of targets like TNF, as revealed through drexml, illustrate the complexity of biological systems and the importance of considering pathway-specific impacts in drug repositioning efforts. Such insights are invaluable to refine drug target predictions and understanding the molecular foundations of diseases such as Fanconi Anemia.

#### 4.1.2 DTSEA: familial melanoma

Analyzing potential drug repurposing opportunities for Familial Melanoma (FM) revealed 8.8% overlap and 14.3% complementary overlap between drug targets identified by DTSEA and drexml.

EGFR stands again as a highly ranked overlapping target. The dual focus on EGFR, targeted by EGFR inhibitors such as Alvocidib and CUDC-101, reflects its critical role in cancer biology, particularly in signaling pathways that drive tumor growth and survival. The EGFR ranking in both approximations highlights its potential as a candidate for FM treatment, suggesting that interventions aimed at this receptor could impact the course of the disease.

The voltage-dependent calcium channel subunit CACNA2D1, associated with drugs such as Pregabalin and Gabapentin enacarbil, also stands out as significantly distant from the disease. The high NES score of these drugs indicates a concentrated interest in modulating calcium channel activity as a therapeutic avenue. Given the role of calcium channels in cellular processes, including proliferation and apoptosis, targeting CACNA2D1 offers a promising strategy for influencing FM pathology, as highlighted in previous research [68].

Additional targets such as LDHB, which is targeted by Artenimol, and PDGFRB, targeted by Trapidil, Erdafitinib, and Ilorasertib, present promising therapeutic options for Familial Melanoma. These drugs, commonly used or in trials for advanced cancers and solid tumors, indicate various mechanisms within pathways relevant to FM. This, combined with the variability in target rankings among the pathways, underscores the intricate nature of the disease's progression.

The combined analysis of DTSEA and drexml highlights the complexity of FM's molecular landscape while identifying key targets and drugs. EGFR and CACNA2D1 emerge as central players in the disease network with their high ranking and significant DREXML scores. Targeting these genes offers the potential for therapeutic intervention to revert or mitigate disease traits in Familial Melanoma.

The analysis further highlights the importance of considering the network context and mechanistic insights when evaluating drug repositioning opportunities. By focusing on genes that play a pivotal role in different signaling circuits, as indicated by the DREXML scores, researchers can prioritize targets that are likely to have a substantial impact on disease progression.

See Table 2 for a summary table of the intersection of both approaches.

### 4.2 CMap analysis

The Connectivity Map (CMap) stores gene expression profiles generated by exposing cells to various agents (referred to as *perturbagens*). It compares these profiles to disease states, identifying genes with similar expression changes, suggesting potential drug targets. CMap assigns each *perturbagen* a connectivity score reflecting its similarity to the disease signature.

#### 4.2.1 CMap: fanconi anemia

When examining all *perturbagen* signatures, 90 out of the 93 targets identified by drexml are included as targets in CMap. Among these, 33% of the targets selected by drexml are located within the top or bottom 5% of the *perturbagen* targets identified in the CMap results. When focusing solely on *perturbagen* signatures derived from chemical compounds, 69 out of the 93 targets selected by drexml are featured as targets in CMap. Among these, 17% of the targets identified by drexml fall within the top or bottom 5% of *perturbagen* targets in the CMap results.

Interestingly, both analyses identified EGFR and PIK3CG as promising candidates for Fanconi Anemia treatment. These targets show high connectivity scores on CMap, suggesting that they can potentially reverse disease-associated gene expression patterns. In addition, the specific circuits affected by their inhibitors and their high DREXML scores indicate targeted therapy with minimal off-target effects. This specificity, evident in the limited number of circuits affected by their inhibitors, makes them strong candidates for further investigation. This is further reinforced by the fact that both genes are highly up-regulated in several tumorigenesis processes derived from impaired DNA repair function in FA patients [69]. This reversal is indicative of a potential therapeutic effect, where *perturbagens* can antagonize the molecular drivers of the disease.

See Table 3 for a summary table of the intersection of both approaches.

**Table 3:** DREXML merged CMAP Comparison of overlapping relevant drugs and relevant targets for FA and FM from the top 5% connectivity score (cmap score) Perturbagen molecules influencing each disease trait.

Target	Perturbagen ID	Perturbagen Name	Mechanism of Action	CMap score	DREXML	Circuits	Dis.
EGFR	BRD-K73293050	WZ-3146	EGFR inhibitor	-2.154	0.002	4	FA
HMGCR	BRD-K22134346	simvastatin	HMGCR inhibitor	-2.148	0.004	1	FA
BTK	BRD-A35869383	ibrutinib	BTK inhibitor	-2.141	0.158	77	FA
PIK3CG	BRD-K42191735	buparlisib	PI3K inhibitor	-2.140	0.001	1	FA
CA12	BRD-K30466858	ellagic-acid	Glutathione transferase inhibitor—Aldose reduct	-2.130	0.004	1	FA
SYK	BRD-K30466858	ellagic-acid	Glutathione transferase inhibitor—Aldose reduct	-2.130	0.044	41	FA
SCN8A	BRD-A53952395	prilocaine	Anesthetic - local	1.899	0.112	36	FA
DGAT2	BRD-K60237333	niacin	NADPH inhibitor—Vitamin B	1.947	0.030	8	FA
HCAR3	BRD-K60237333	niacin	NADPH inhibitor—Vitamin B	1.947	0.103	70	FA
NNMT	BRD-K60237333	niacin	NADPH inhibitor—Vitamin B	1.947	0.032	10	FA
ALOX5	BRD-A87479750	tenidap	Cyclooxygenase inhibitor	1.952	0.170	83	FA
HCAR2	BRD-K92428153	mycophenolate-mofetil	Inosine monophosphate dehydrogenase inhibitor	2.083	0.183	87	FA
EDNRA	BRD-K11433652	aspirin	Cyclooxygenase inhibitor	-1.971	0.006	1	FM
NFKB1	BRD-K11433652	aspirin	Cyclooxygenase inhibitor	-1.971	0.708	30	FM
KCNJ8	BRD-K73109821	diazoxide	Potassium channel activator	-1.931	0.113	9	FM
AR	BRD-K41494493	bisphenol-a	Synthetic estrogen	-1.922	0.005	1	FM
CDK4	BRD-K41564320	purvalanol-b	CDK inhibitor	1.954	0.610	34	FM
EGFR	BRD-K88741031	BRD-K88741031	Tyrosine kinase inhibitor—EGFR inhibitor	1.981	0.170	14	FM
KDR	BRD-K63504947	semaxanib	VEGFR inhibitor	1.996	0.007	1	FM
PDGFRA	BRD-K93918653	quizartinib	FLT3 inhibitor	2.044	0.012	1	FM
PDGFRB	BRD-K93918653	quizartinib	FLT3 inhibitor	2.044	0.024	3	FM
TYR	BRD-K03981224	ethisterone	Progestogen hormone	2.069	0.045	3	FM
SCNN1A	BRD-K92049597	triamterene	Sodium channel inhibitor	2.163	0.011	1	FM

#### 4.2.2 CMap: familial melanoma

Familial Melanoma, a genetically predisposed form of melanoma, presents unique challenges and opportunities for targeted therapy. The CMap comparison with drexml highlights several key targets and the corresponding *perturbagens* with notable connectivity scores, suggesting their potential efficacy in reversing disease-specific gene expression patterns. Some of the highest-ranked targets, in terms of Connectivity scores (CMap Scores), that count with specific and impactful DREXML scores across the FM disease map are: CDK4, TYR, EGFR, and NFKB1.

Although certain *perturbagen* molecules, such as aspirin, do not exhibit the potential to revert the disease progression, it highlights the activity of inflammatory processes to which NFKB1 is a central regulator [51]. In contrast, EGFR emerges as a promising target, with tyrosine kinase inhibitors offering a targeted approach to modulate key pathways implicated in melanoma progression. The specificity of these inhibitors, as evidenced by the selective number of circuits they impact, while obtaining a high connectivity score. This targeted approach aligns with the current trend in cancer therapy, focusing on disrupting specific signaling pathways critical to tumor growth and survival.

The targeting of TYR by Ethisterone introduces a novel therapeutic perspective, exploring the role of hormonal modulation in melanoma treatment. Despite the high connectivity score indicating potential efficacy, the minimal number of circuits affected by the drexml results highlights the specificity of this approach. Although innovative, this avenue calls for a rigorous investigation to fully understand the implications of hormonal pathway modulation in melanoma therapy. The latest research shows that high androgen receptor activity is required for melanoma cell proliferation and tumorigenesis [70].

The identification of CDK4 as a key target in the context of Familial Melanoma underscores the critical role of cell cycle dysregulation in cancer progression. Purvalanol-b, acting as a CDK inhibitor, shows significant potential to disrupt this process. The connectivity score from the CMap analysis, as shown in Table 3, suggests its efficacy in reversing melanoma-specific gene expression patterns. However, the broad impact across numerous biological circuits requires careful evaluation of potential off-target effects, emphasizing the importance of precision in its therapeutic application.

Overall, our comparative analysis from CMap and drexml insights, not only identifies promising targets but also emphasizes the nuanced understanding required to navigate the complex landscape of therapeutic interventions. The complementarity of both approaches highlights the importance of gathering several layers of evidence to successfully search for new therapeutic options.

## 5 Impact

In the past, a preliminary version of the DREXML methodology was used in [40] to repurpose drugs for Fanconi Anemia. However, the initial version of the model did not have per-circuit relevance scores, stability measures, or fair feature attributions, making it difficult to interpret biologically. Despite these limitations, some of the highest-ranked drug targets predicted by the algorithm were experimentally validated in [49] with positive results. In subsection 3.1 we show the usefulness of the improved DREXML methodology provided in the drexml package: as the explainability of the methodology has increased, so does the ability of the end user to assert if certain biological and medical constraints are met.

To further demonstrate the utility of our method for drug repurposing in rare diseases, we present the first results of using the drexml package to identify repurposable drugs for familial melanoma (see subsection 3.2). Furthermore, the methodology has been used to successfully repurpose drugs for retinitis pigmentosa, resulting in the experimental validation in mice of several drug targets [71]. Furthermore, the methodology was central to our search for repurposable drugs for COVID-19 [72], demonstrating its ability to contextualize protein drug targets in terms of the functional landscape of the disease, demonstrating the generality of the methodology beyond rare diseases.

Furthermore, we performed a comparative analysis with two leading drug repurposing tools: the Connectivity Map (CMap) and Drug Target Enrichment Set Analysis (DTSEA). These three tools offer complementary perspectives on the druggable landscape for Familial Melanoma and Fanconi Anemia. Notably, despite employing distinct methodologies, all three tools converge on several key targets, bolstering the evidence-based selection of candidates for future experimentation.

In summary, the drexml package is a modernized version of a drug repurposing approach that was never implemented in software form. Despite its lack of formal software implementation, the methodology was highly successful, resulting in multiple studies [40], [72] that identified potential drug repurposing candidates through both laboratory [49], [71] and real-world data [73], [74]. We expect that the availability of the drexml package will encourage more people to use this methodology, thus expanding the druggable space for various diseases and furthering research in this area.

## 6 Conclusions

The drexml package offers a convenient way to repurpose drugs for any given disease. When biological or medical constraints are taken into account, it can be used to validate hypotheses, such as the Fanconi Anemia and Familial Melanoma cases discussed in this work. Even with limited prior knowledge, the tool can be used to filter and rank repurposable drugs, while providing an understanding of their effect on the disease, as seen in the case of emerging diseases like COVID-19.

## CRediT authorship contribution statement

**Marina Esteban-Medina**: Methodology, Software, Validation, Visualization, Formal analysis, Data curation, Writing – original draft. **Víctor Manuel de la Oliva Roque**: Software, Validation, Formal analysis, Writing – original draft & documentation. **Sara Herráiz-Gil**: User experience, Writing – original draft & documentation. **María Peña-Chilet**: Methodology, Conceptualization, Funding acquisition, Writing – review. **Joaquín Dopazo**: Methodology, Conceptualization, Funding acquisition, Writing – review. **Carlos Loucera**: Methodology, Conceptualization, Software, Supervision, Funding acquisition, Writing – original draft, review & editing.

## Declaration of Competing Interest

The authors declare no conflicts of interest.

## Data Availability

All data are publicly available at the Zenodo data repository https://zenodo.org/doi/10.5281/zenodo.6020480. All datasets are automatically downloaded when using the package for the first time. The software is freely available under the MIT license at https://github.com/loucerac/drexml and on the Python Package Index at https://pypi.org/project/drexml/. A permanent copy is stored at Zenodo https://doi.org/10.5281/zenodo.10715262.
